# Immediate risk of cervical intraepithelial neoplasia and diagnostic value of colposcopy among cytology-negative women with oncogenic HPV: a retrospective study

**DOI:** 10.1186/s12905-024-03258-x

**Published:** 2024-07-24

**Authors:** Weichao Dai, Tongfei Wang, Lin Chen, Zhongyuan Qiu, Peifang Chen, Dezhao Chen

**Affiliations:** Department of Gynaecology and Obstetrics, Fujian Medical University Union Hospital, Fuzhou, 350001 Fujian Province China

**Keywords:** Cervical cancer screening, Cervical dysplasia, Cervical biopsy, Diagnostic value

## Abstract

**Background:**

Cervical cancer screening results that are negative for cytology but positive for high-risk human papillomavirus (HR-HPV) are not uncommon. One-year follow-up is suggested for patients with no history of HPV positivity under the most recent American Society of Colposcopy and Cervical Pathology (ASCCP) guidelines (2019). The aim of this study was to evaluate the immediate risk of cervical intraepithelial neoplasia (CIN) among cytology-negative patients positive for HR-HPV. The diagnostic accuracy of colposcopy in these patients was investigated.

**Methods:**

A retrospective study was conducted in patients who were cytology negative but HR-HPV positive and referred for colposcopy from January 2022 to August 2023. Patients were compared in terms of the immediate rate of CIN lesions among the HPV16-positive group, the HPV18-positive group and the non-16/18 HR-HPV-positive group. The distribution of CIN2 + lesions according to age was evaluated. The factors associated with the accuracy of colposcopy were evaluated using univariate and multivariate logistic regression.

**Results:**

Among the 372 patients, 195 had chronic cervicitis, 131 had CIN1, 37 had CIN2/3, and nine had carcinoma. The immediate rates of CIN2 + lesions and CIN3 + lesions in patients who were not HR-HPV16/18-positive were comparable to those in patients who were HPV16/18-positive (*P* = 0.699). In addition, among patients diagnosed with CIN2 + lesions, 8 (17.39%) patients were women aged < 30 years. When pathological results were used as a reference, the consistency rate of colposcopy was 61.0% (227/372). Multivariate analyses revealed that age and the type of cervical transformation zone were independent factors affecting the accuracy of colposcopy (*P* < 0.001).

**Conclusions:**

In countries with limited resources, immediate colposcopy referral should be recommended for patients who are cytology negative but HR-HPV-positive (including non-16/18 HR-HPV-positive), and cervical cancer screening via cotesting should be suggested for women aged < 30 years. Colposcopy has moderate diagnostic value and can be affected by age and the type of cervical transformation zone.

## Background

Cervical cancer, which is the third most common cancer and the third leading cause of cancer death in females, poses a serious threat to women’s health [1, 2]. In China, the incidence and mortality of cervical cancer are 7.5/100,000 and 3.4/100,000, respectively [3]. Thus, it remains an important public health problem.

Persistent infection with high-risk human papillomavirus (HR-HPV) plays a crucial role in causing cervical intraepithelial neoplasia (CIN) and cervical cancer [4, 5]. It often takes 10–15 years for CIN to develop into cervical cancer after persistent infection with HR-HPV [6, 7]. Thus, it provides many opportunities to detect and treat precancerous lesions. Cervical cytology, which is a routine and widely used cervical cancer screening method, has a sensitivity of only approximately 50% [5, 8]. In contrast, HPV-based screening provides 60-70% greater protection against cervical cancer [9]. Due to the relatively low sensitivity of cervical cytology, discordant cotesting, which is defined as being cytology negative but HR-HPV-positive, is common when using combined screening [10, 11]. However, managing women with discordant cotesting remains a challenge.

An estimated 12% of cytology-negative women have HR-HPV infection [11]. In addition, approximately 89% of cervical cancer patients are HR-HPV-positive [11]. According to the current guidelines [10, 12], approximately 10% of cervical high-grade lesions caused by HR-HPV other than HPV16 and 18 might be missed, particularly in countries with limited professional cytologists [13, 14]. Additionally, managing women with such cases is controversial. Owing to the low risk of immediate cancer for patients who are cytology negative but HR-HPV-positive, excessive intervention or overreferral to colposcopy are not recommended [15].

Based on these observations, the use of colposcopy might help identify precancerous lesions and cervical cancer in these patients. In addition, unnecessary invasive cervical biopsies might be reduced if colposcopy is appropriately used. Therefore, this study aimed to explore the discrepancies between colposcopy diagnosis results and cervical biopsy results in diagnosing precancerous lesions and to investigate the immediate risk of CIN in patients with cytology-negative but HR-HPV-positive results.

## Methods

### Study population

This retrospective study included women who underwent HPV tests and liquid-based cytology (LBC) tests for cervical screening and who were referred for colposcopy examination between January 2022 and August 2023 at Fujian Medical University Union Hospital, Fuzhou, China. The following patients were eligible for inclusion in this study: (1) women who were older than 16 years and had a history of sexual activity, (2) patients who tested positive for HR-HPV and negative for the LBC test, (3) patients who underwent colposcopy-guided biopsy once they tested positive for HR-HPV, and (4) patients who had pathological resuslts of biopsy tissue. The exclusion criteria were as follows: (1) pregnant; (2) had a history of surgical treatment for CIN or cervical cancer, radiotherapy or chemotherapy; (3) had undergone colposcopy but without cervical biopsy; and (4) had a history of persistent or transient HR-HPV infection. To mitigate the impact of posttreatment pathological result reversals, we opted to rely on the initial colposcopy findings.

The study was approved by the Medical Ethics Committee of Fujian Medical University Union Hospital (Date15/1/2020/No2020QH023). Informed consent was obtained from all participants before the study.

### Examination method

The LBC test was used to perform cervical cytology analysis. The results were interpreted by experienced pathologists and classified according to the 2014 Bethesda System [16]. Cervical cytology negative indicated no intraepithelial lesions or malignancy (NILM). HPV genotypes were examined by an HPV GenoArray test kit (HybriBio Ltd), which is capable of identifying seventeen HR-HPV types (16, 18, 31, 33, 35, 39, 45, 51, 52, 53, 56, 58, 59, 66, 68, 73, and 82) and six low-risk HPV types (6, 11, 42, 43, 81, and 83). Patients infected with HR-HPV other than HPV16/18 were classified into the “non-16/18 HR-HPV” group.

Colposcopic examinations were conducted in accordance with the 2011 International Federation of Cervical Pathology and Colposcopy (IFCPC) [17] by different colposcopists. Senior colposcopists were defined as colposcopists who had more than ten years of working experience, while others were defined as junior colposcopists. Multipoint cervical biopsies were taken at abnormal imaging sites. When no suspicious lesions were observed, routine biopsy was performed at points 3, 6, 9 and 12. Endocervical curettage (ECC) was performed when the lesions had spread into the cervical canal or were not fully visible.

The pathological biopsy results included chronic cervicitis, CIN1, CIN2, CIN3 and invasive carcinoma. The colposcopy results included a normal impression, a low-grade impression, and a high-grade impression (including carcinoma). If colposcopy suggested a normal impression and biopsy suggested chronic cervicitis, the two test results were considered consistent. If colposcopy suggested a low-grade lesion and biopsy suggested CIN1, as well as when colposcopy suggested a high-grade lesion and biopsy suggested CIN2, CIN3 or invasive carcinoma, the two test results were considered consistent. Otherwise, the two tests were considered inconsistent.

## Statistical analysis

Data analysis was performed using SPSS software version 25.0. Normally distributed data are presented as the means ± standard deviations (mean ± SD). Categorical data are expressed as n (%) and were analysed using the chi-square test. Univariate and multivariate logistic regression analyses were used to evaluate independent factors associated with the outcomes. A P value < 0.05 was considered to indicate statistical significance.

## Results

The characteristics of the patients included in this study are presented in Table 1. In total, 372 patients who were cytology negative but HR-HPV-positive and who underwent colposcopy and cervical biopsy were included in the study. The included patients ranged in age from 16 to 79 years. Of the patients, 168 (45.16%) had HPV16 infection, 98 (26.34%) had HPV18 infection, and 106 (28.5%) had non-HPV16/18 infection. Pathology revealed 195 (52.42%) chronic cervicitis patients, 131 (35.21%) CIN1 patients, 37 (9.95%) CIN2/3 patients and nine (2.42%) invasive carcinoma patients. Normal colposcopy impressions were the most common colposcopy diagnosis (47.31%), followed by low-grade impressions (43.82%) and high-grade impressions (8.87%).

**Table 1:** Summary of clinical characteristics among the study participants

Characteristics	Number	Percentage(%)
Total	372	
Age (mean ± SD), years old	44.0 ± 11.8	
HR-HPV typing		
HR-HPV16	168	45.16
HR-HPV18	98	26.34
Non-16/18 HR-HPV	106	28.50
Pathological biopsy result		
Chronic cervicitis	195	52.42
CIN1^a^	131	35.21
CIN2/3^a^	37	9.95
Invasive carcinoma	9	2.42
Colposcopy diagnosis result		
Normal impression	176	47.31
Low grade impression	163	43.82
High grade impression	33	8.87
Transformation zone		
Type I cervical transformation zone	64	17.20
Type II cervical transformation zone	112	30.11
Type III cervical transformation zone	196	52.69
Colposcopist’s skills		
Junior	271	72.85
Senior	101	27.15

^a^ CIN1: cervical intraepithelial neoplasia grade 1; CIN2/3: cervical intraepithelial neoplasia grade 2/3

The consistency between the pathological biopsy results and colposcopy diagnosis results is depicted in Table 2. With pathological biopsy results as the gold standard, 227 (61.02%) patients had consistent colposcopy results. Among patients with CIN2 + lesions (including CIN2, CIN3 and invasive carcinoma), the accuracy was high (91.1%), and the sensitivity was 50.0%, while the specificity was 96.9%. With respect to normal colposcopy images, the accuracy was 67.5%, the sensitivity was 64.1%, and the specificity was 71.2%. The sensitivity for detecting CIN1 was 60.3%, the specificity was 65.1%, and the accuracy was 63.4%.

**Table 2:** Correlations between pathological biopsy results and colposcopy diagnosis results

	Pathological biopsy results					
Colposcopy diagnosis results	Chronic cervicitis *N* = 195(%)	CIN1^a^ *N* = 131(%)	CIN2 + ^a^ lesions *N* = 46(%)	Sensitivity	Specificity	Accuracy
Normal impression	125(71.02%)	47(27.84%)	4(2.27%)	64.1%	71.2%	67.5%
Low grade impression	65(39.88%)	79(48.46%)	19(11.66%)	60.3%	65.1%	63.4%
High grade impression	5(15.15%)	5(15.15%)	23(69.70%)	50.0%	96.9%	91.1%

^a^ CIN1: cervical intraepithelial neoplasia grade 1; CIN2+: cervical intraepithelial neoplasia grade 2 or worse

The associations between the clinical factors and the accuracy of colposcopy diagnosis according to univariate and multivariate logistic regression analyses are presented in Table 3. Since 48 years of age is the general age at which individuals enter perimenopause in China, the effect was analysed before and after 48 years of age [18]. Univariate analysis revealed that the type of cervical transformation zone affected the accuracy of the colposcopy diagnosis (*P* < 0.001). Multivariate analysis demonstrated that age and the type of cervical transformation zone affected the accuracy of the colposcopy diagnosis (*P* = 0.028 and < 0.001, respectively). HPV genotype and colposcopist skill had no significant effect on the accuracy of the colposcopy diagnosis.

**Table 3:** Univariate and multivariate analyses of colposcopy accuracy

Index	Univariate analysis			Multivariate analysis		
	OR	95%CI	*P*	OR	95%CI	*P*
Age						
< 48 years old	1.030	0.676–1.568	0.892	0.570	0.345–0.941	0.028
≥ 48 years old						
Transformation zone						
Type I/II	0.430	0.281–0.658	< 0.001	2.995	1.832–4.896	< 0.001
Type III						
HPV type						
HPV16/18	1.204	0.755–1.920	0.435	0.818	0.501–1.334	0.420
Non-16/18 HR-HPV^a^						
Colposcopist’s skills						
Junior	0.823	0.512–1.322	0.421	1.377	0.842–2.250	0.202
Senior						

^a^ HR-HPV: high-risk human papillomavirus

According to the pathological biopsy results, the percentages of immediate CIN2 + lesions in patients with HPV16 infection, HPV18 infection and non-HPV16/18 infection were 15.48%, 8.16% and 11.32%, respectively (*P* = 0.202), while the percentages of immediate CIN3 + lesions were 5.95%, 8.16% and 2.83%, respectively (*P* = 0.263)(Table 4). The immediate rates of invasive carcinoma in patients with HPV16 and HPV18 infections were 2.38% and 5.10%, respectively (*P* = 0.405). No cases of carcinoma were found in the non-16/18 HR-HPV group.

**Table 4:** Comparison of high-risk HPV genotypes and pathological results

Pathological biopsy results	HPV16 (*N* = 168)	HPV18 (*N* = 98)	Non-16/18 HR-HPV^a^ (*N* = 106)	*P*
Chronic cervicitis	84(50%)	54(55.10%)	57(53.77%)	0.686
CIN1^a^	58(34.52%)	36(36.74%)	37(34.91%)	0.933
Invasive carcinoma	4(2.38%)	5(5.10%)	0(0)	0.060
CIN2 + ^a^ lesions	26(15.48%)	8(8.16%)	12(11.32%)	0.202
CIN3 + ^a^ lesions	10(5.95%)	8(8.16%)	3(2.83%)	0.263

^a^ CIN1: cervical intraepithelial neoplasia grade 1; CIN2+: cervical intraepithelial neoplasia grade 2 or worse; CIN3+: cervical intraepithelial neoplasia grade 3 or worse; HR-HPV: high-risk human papillomavirus

No significant differences were found when comparing the immediate incidence of CIN2 + lesions between the HPV16/18 group and the non-HPV16/18 HR-HPV group (*P* = 0.699). Comparisons of the percentages of patients with CIN2 + lesions in the < 30 years, 30–39 years, 40–49 years, and ≥ 50 years age groups are shown in Table 5. In the < 30 years age group, statistical evaluation could not be performed because there were too few patients in the non-16/18 HR-HPV-positive subgroup, and no patients with CIN2 + lesions were found in this subgroup (0/4). In the 40–49 years age group, the percentage of CIN2 + lesions was significantly greater in the non-16/18 HR-HPV group than in the HPV16/18 group (*p* = 0.022). No significant differences were found in the 30–39 years age group or the ≥ 50 years age group.

**Table 5:** The age-stratified distribution of pathological cervical intraepithelial neoplasia grade 2 or worse in the study groups

CIN2 + ^a^	Study group		
Age group (years)	HPV16/18 *N*(%)	Non-16/18 HR-HPV^a^ *N*(%)	*P*
< 30	8/36(22.22)	0/4(0)	-
30–39	9/78(11.54)	2/22(9.09)	0.746
40–49	3/64(4.69)	6/31(19.35)	0.022
≥ 50	14/88(15.91)	4/49(8.16)	0.198
Total	34/266(12.78)	12/106(11.32)	0.699

^a^ CIN2+: cervical intraepithelial neoplasia grade 2 or worse; HR-HPV: high-risk human papillomavirus

**Fig. 1:**
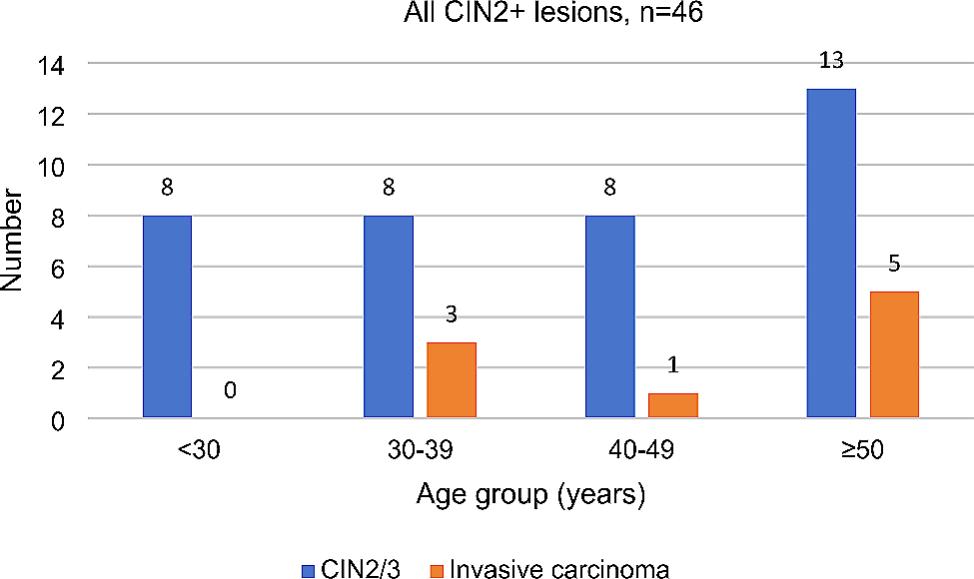
Distribution of pathological cervical intraepithelial neoplasia grade 2 or worse lesions according to age. CIN2/3: cervical intraepithelial neoplasia grade 2/3; CIN2+: cervical intraepithelial neoplasia grade 2 or worse

When the patients diagnosed with CIN2 + lesions were evaluated according to age group, as demonstrated in Fig. 1, there were 8 (17.39%) patients in the < 30 years age group. In the < 30 years age subgroup, 5 patients were aged < 25 years, and 3 patients were aged 25–29 years. In regard to invasive carcinoma, there were 3 cases, 1 case and 5 cases in the 30–39 years age group, 40–49 years age group and ≥ 50 years age group, respectively. However, no cases of carcinoma were found in the < 30 years age group.

## Discussion

Cervical cytology, HPV testing, and colposcopy are screening methods capable of detecting cervical dysplasia and early-stage cervical carcinoma. These diagnostic tools help guide patients towards appropriate management modalities and subsequent follow-up, ensuring timely intervention and care [2]. Recently, studies have shown that cytology-negative but HR-HPV-positive results are common when using combined screening tests, and the rates of high-grade CIN and cervical cancer are significantly greater in women who are cytology-negative but HR-HPV-positive than in those who are cytology-negative only [10, 11, 19]. However, the best management for women who are cytology negative but HR-HPV-positive, particularly those who are non-16/18 HR-HPV-positive, is still controversial. Colposcopy is reported to be the main procedure and cost-effective examination for accurate diagnosis of high-grade CIN [20]. Nevertheless, in clinical practice, the consistency and accuracy of colposcopy for diagnosing high-grade squamous intraepithelial lesion (HSIL) still need to be improved. Based on these observations, the immediate risk of CIN in patients who were cytology negative but HR-HPV-positive and the diagnostic accuracy of colposcopy were investigated in this study.

In this study, of the 372 patients who were cytology negative but HR-HPV-positive, 131 had CIN1, 37 had CIN2/3, and nine had carcinoma. The percentages of CIN2 + lesions and CIN3 + lesions in patients who were not HR-HPV 16/18-positive were comparable to those in patients who were HPV16- or HPV18-positive. In addition, among patients diagnosed with CIN2 + lesions, 8 (17.39%) patients were women aged < 30 years. When pathological results were used as a reference, the consistency rate of colposcopy was 61.0% (227/372). These findings suggested that, in countries with limited resources, it is feasible to recommend immediate colposcopy referral in patients who are cytology negative but HR-HPV-positive (including non-16/18-positive patients). In addition, cervical cancer screening by cotesting in women aged < 30 years should be suggested in countries with poor resources.

Previous studies have shown that approximately 1.9–9.8% of women over 30 years old are cytology negative but HR-HPV-positive [10]. Studies have demonstrated that the 1-year risk of CIN3 + lesions is less than 4%, while the 5-year risk of CIN3 + lesions is 6.4% in these patients [21, 22]. Follow-up after 1 year is suggested for patients with no history of HPV positivity according to the most recent American Society of Colposcopy and Cervical Pathology (ASCCP) guidelines (2019) [23]. The immediate risks of CIN2 + lesions and CIN3 + lesions were 4.99% and 2.13%, respectively, in these patients [24]. In addition, in women who were HPV16-positive but cytology-negative, the immediate risks of CIN2 + lesions and CIN3 + lesions were 7.82% and 5.30%, respectively. For patients who were HPV18-positive but cytology-negative, the immediate risks of CIN2 + lesions and CIN3 + lesions were 5.56% and 3%, respectively [24]. The immediate risk of CIN2 + lesions for patients who were non-16/18 HR-HPV-positive and cytology-negative ranged from 1.1% to 6.5% [25–27]. In this study, the percentages of patients with immediate CIN2 + lesions were 15.48%, 8.16% and 11.32% among cytology-negative patients who were HPV16-positive, HPV18-positive and non-16/18 HR-HPV-positive, respectively (*P* = 0.202), while the percentages of patients with immediate CIN3 + lesions were 5.95%, 8.16% and 2.83%, respectively (*P* = 0.263). Hence, this study demonstrated that the incidence of CIN2 + lesions and CIN3 + lesions in non-16/18 HR-HPV-positive patients was comparable to that in HPV16- or HPV18-positive patients. This result is consistent with the studies of Kabaca et al. [26] and Athena HPV study group. [28]. When referred to invasive carcinoma, the percentages were 2.38%, 5.10% and 0% among cytology-negative patients who were HPV-16 positive, HPV-18 positive and non-16/18 HR-HPV-positive in this study, respectively (*P* = 0.060). The present study did not find cervical carcinoma in patients with cytology-negative but non-16/18 HR-HPV-positive, which was in consistent with previous studies [27, 29]. This result might be attributed to the limited sample size and relatively low incidence of carcinoma in this particular patient cohort. As reported by Kabaca et al., only one (0.1%) (HPV-39 positive) of seven hundred and fifty-two patients with cytology-negative but non-16/18 HR-HPV-positive had invasive carcinoma [26]. Zappacosta et al. reported a case of HPV-53-related cervical cancer in a 79-year-old woman with cytology-negative, which might be due to the underutilization of screening methods and the low sensitivity of cervical cytology test [30]. Besides, before the publication of Zappacosta et al.’s findings, HPV53 infection had never been reported in patients with cervical cancer. These researches have suggested that despite the low incidence of cervical carcinoma, carcinoma has indeed been detected in patients with cytology-negative but non-16/18 HR-HPV-positive. Recently, HPV31/33/39 genotyping and multiple HPV31/32/52 infections were proposed to be added to the previous recommended HPV16/18 genotyping triage for colposcopy [26, 31, 32]. Based on the aforementioned observations that the incidence of CIN2 + lesions and CIN3 + lesions in non-16/18 HR-HPV-positive patients was comparable to that in HPV16- or HPV18-positive patients, along with the detection of cervical carcinoma in non-16/18 HR-HPV-positvie patients, it could be feasible to perform colposcopy in cytology-negative patients who are non-16/18 HR-HPV-positive, as in those who are HPV16/18-positive. The implementation of repeated screening by cotesting in women who are cytology negative but HR-HPV-positive remains a considerable challenge in limited resource areas such as China. Thus, regardless of the low immediate risk of CIN3 + lesions in HR-HPV-positive and cytology-negative patients, the recommendation of immediate colposcopy referral should be suggested for these patients.

Although cervical cancer is rare in women younger than 30 years, an increasing trend in the incidence of cervical cancer has been demonstrated in young women [33]. Gumpeny N et al. reported that 13.2% of pathologically diagnosed CIN2 + lesions (including carcinoma) were observed in women aged 21–30 years [34]. Tidy JA et al. reported that 14.2% of CIN2 + lesions were detected in women aged 25–34 years who were cytology-negative but persistently HR-HPV-positive [35]. In this study, 17.39% (8/46) of CIN2 + lesions were detected in cytology-negative women aged < 30 years with HPV16/18 positivity, and 10.87% (5/46) of CIN2 + lesions were detected in women aged < 25 years. Although some cases of CIN2 might regress spontaneously in young women, a large cohort study of pathological results of CIN showed that 34.6% of patients in the 25- to 30-year-old age group experienced persistent disease, and 13.1% of patients in the 25- to 30-year-old age group experienced lesion progression [36]. Approximately 36% of women < 25 years with CIN2 would experience persistent disease without treatment within 2 years, while the percentage would increase to 69% among those who were HPV16-positive [37]. A retrospective survey of a sample of 2966 patients who underwent conization for high-grade cervical lesions revealed that patients with 12-month HPV persistence had a 2-fold greater 5-year recurrence rate than did those with 6-month HPV persistence [38]. The crude recurrence rates were approximately 7.46%, 13.1%, and 10.3% for patients with 6-, 12-, and 24-month HPV persistence, respectively [2, 38, 39]. These studies shed light on the fact that high-grade cervical lesions do occur in women < 30 years of age, including those who are cytology negative but HR-HPV-positive, as well as the potential impact of HPV persistence on long-term outcomes. Patients < 30 years of age with CIN2 + lesions are likely to be missed or progress to malignancy if they are screened by cervical cytology alone according to the American College of Obstetricians and Gynaecologists [40] and the United States Preventive Services Task Force [41] recommendation or are managed under the most recent ASCCP guidelines (2019) [23]. Thus, cervical cancer screening by cotesting in women aged < 30 years should be suggested, and immediate colposcopy referral should be recommended.

As a subjective examination method, colposcopy can detect more CIN lesions. Studies have shown that the accuracy of colposcopy can be influenced by several factors, such as the expertise of the colposcopist, HPV genotype, HPV viral load, cytology results, transformation zone type and age [42, 43]. The overall accuracy of colposcopy in detecting CIN2 + lesions ranges from 69.7% to 89%, the sensitivity ranges from 30% to 90%, and the specificity ranges from 44% to 97% [20, 44–46]. This study demonstrated a comparable accuracy of colposcopy diagnosis (91.1%), with relatively lower sensitivity (50.0%) and increased specificity (96.9%) when CIN2 + lesions was the threshold. Moreover, this study suggested that age and the type of cervical transformation zone were the factors affecting the accuracy of colposcopy diagnosis, which is consistent with the findings of Liu et al. [47]. This may be due to low oestrogen levels and incomplete exposure of the cervical transformation zone. Notably, nearly half of the CIN2 + lesions patients were missed at initial colposcopy based on the results of this study and Bangladesh’s results [48]. Therefore, in the event of a suspicious colposcopy result, age and the transformation zone should be taken into consideration along with clinical symptoms to reduce the risk of missed cervical lesions.

## Conclusions

In conclusion, this study revealed that the percentages of immediate CIN2 + lesions and CIN3 + lesions in cytology-negative patients who were non-16/18 HR-HPV-positive were comparable to those who were HPV16/18-positive. Follow-up after 1 year might be unsafe for these patients, as a majority of the screening patients would have insufficient follow-up in countries such as China. High-grade cervical lesions occur in women < 30 years of age, including those who are cytology negative but HR-HPV-positive. Thus, it is feasible to recommend immediate colposcopy referral in patients who are cytology negative but HR-HPV-positive (including non-16/18 positive) in countries with limited resources. In addition, cervical cancer screening by cotesting in women aged < 30 years should be suggested in countries with poor resources. Colposcopy has moderate diagnostic value and can be affected by age and the type of cervical transformation zone.

The strength of the study is the use of total genotyping, which allowed us to also study the frequency of non-16/18 HPV genotypes in CIN2 + lesions. This is important because it has been demonstrated that CIN2 + lesions are frequently associated with non-16/18 HPV genotypes in older women. This study had several limitations. First, this was a retrospective study with a small sample size. We anticipate that larger sample sizes and prospective, randomized, and multicentre research findings will yield a better clinical assessment of patients and a better choice in terms of the type of therapy. Second, it was difficult to determine whether the influencing factors were independently associated with outcomes in this retrospective study. Third, long-term follow-up could not be estimated in this study because the study only evaluated the immediate risk of CIN2 + lesions, and whether the duration of HPV persistence might impact the risk of recurrence could not be evaluated. The long-term risk of CIN2 + lesions during follow-up and the risk of recurrence in patients with persistent HPV might be evaluated in future studies. Finally, as this study was limited by the small sample size of the Chinese population, further research is necessary to ascertain the applicability and generalizability of these findings to other populations.

## Data Availability

The datasets used and/or analysed during the current study are available from the corresponding author on reasonable request.

## Abbreviations

HR-HPV: High-risk human papillomavirus
ASCCP: American Society of Colposcopy and Cervical Pathology
CIN: Cervical intraepithelial neoplasia
LBC: Liquid-based cytology
NILM: No intraepithelial lesions or malignancy
HSIL: High-grade squamous intraepithelial lesion
